# Xylo-Oligosaccharides and Inulin Affect Genotoxicity and Bacterial Populations Differently in a Human Colonic Simulator Challenged with Soy Protein

**DOI:** 10.3390/nu5093740

**Published:** 2013-09-23

**Authors:** Claus T. Christophersen, Anne Petersen, Tine R. Licht, Michael A. Conlon

**Affiliations:** 1 Preventative Health National Research Flagship, CSIRO Animal, Food and Health Sciences, PO Box 10041, Adelaide BC SA 5000, Australia; E-Mail: c.christophersen@csiro.au; 2 National Food Institute, Division of Microbiology and Risk Assessment, Technical University of Denmark, Mørkhøj Bygade 19, Søborg 2860, Denmark; E-Mails: apetersen.mail@gmail.com (A.P.); trli@food.dtu.dk (T.R.L.)

**Keywords:** microbiota, DNA damage, comet assay, microbial fermentation, quantitative PCR

## Abstract

High dietary intakes of some protein sources, including soy protein, can increase colonic DNA damage in animals, whereas some carbohydrates attenuate this. We investigated whether inulin and xylo-oligosaccharides (XOS) could be protective against DNA strand breaks by adding them to a human colonic simulator consisting of a proximal vessel (PV) (pH 5.5) and a distal vessel (DV) (pH 6.8) inoculated with human faeces and media containing soy protein. Genotoxicity of the liquid phase and microbial population changes in the vessels were measured. Soy protein (3%) was fermented with 1% low amylose cornstarch for 10 day followed by soy protein with 1% XOS or 1% inulin for 10 day. Inulin did not alter genotoxicity but XOS significantly reduced PV genotoxicity and increased DV genotoxicity. Inulin and XOS significantly increased butyrate concentration in the DV but not PV. Numbers of the key butyrate-producing bacterium *Faecalibacterium prausnitzii* were significantly increased in the PV and DV by inulin but significantly decreased by XOS in both vessels. Other bacteria examined were also significantly impacted by the carbohydrate treatments or by the vessel (*i.e.*, pH). There was a significant overall inverse correlation between levels of damage induced by the ferments and levels of sulphate-reducing bacteria, *Bacteroides fragilis*, and acetate. In conclusion, dietary XOS can potentially modulate the genotoxicity of the colonic environment and specific bacterial groups and short chain fatty acids may mediate this.

## 1. Introduction

Most large epidemiological studies have shown an association between diets high in red and processed meat and an increased risk of colorectal cancer (CRC) [1]. Experimental studies involving animals indicate that high dietary intakes of these and other sources of protein, including a soy protein isolate, can increase the genotoxicity of the colonic luminal environment resulting in increased colonic DNA damage, whereas dietary fibre is associated with protection against CRC and forms such as resistant starch (RS) and arabinoxylan can help protect against dietary protein-induced colonic damage [1,2,3,4,5]. This protection appears partly related to the ability of many fibres to reach the large bowel and undergo fermentation, resulting in the production of short chain fatty acids (SCFA) and possibly other products which promote colonic tissue integrity. A lowering of levels of genotoxic protein fermentation products and the acidification of the colonic environment may also contribute to the protection. Faecal bulking and reduced stool transit time also reduce exposure of colorectal tissues to potentially toxic colonic agents [6].

Prebiotic carbohydrates, which selectively stimulate the growth and activity of beneficial bacteria in the intestinal tract [7,8], have the potential to help protect against colorectal disease. The best studied of the prebiotics are inulin and fructo-oligosaccharides (FOS), while xylo-oligosaccharides (XOS) are considered as promising prebiotic candidates [7,9]. XOS oligomers are composed of xylose units linked by β-1.4 linkages [2,10]. Due to their β-configuration the oligomers are not degraded by human digestive enzymes and reach the large intestine chemically intact, where they act as a substrate for the colonic microbiota [10]. There is some evidence that inulin could be genoprotective [11]. Also, inulin has been found to increase the abundance of *Faecalibacterium prausnitzii* [12], a key butyrate-producing bacterium with anti-inflammatory properties [13]. The ability to generate butyrate may be important for colonic genoprotection, as the ability of dietary RS to protect against high dietary protein-induced colonic DNA damage in rats is strongly correlated with levels of this SCFA [3]. Acetate may also play a role, as recent evidence suggests it can protect against the toxicity of pathogenic enteric bacteria [14] and has an inverse relationship with DNA damage [3]. However, it is not known if XOS can protect against dietary protein-induced colonic DNA damage.

In the present study, we have used a simulator of the human colon inoculated with human faeces and a soy protein isolate (that can induce relatively high colonic DNA damage when consumed by rats [4]) to examine whether addition of XOS to the simulator can reduce the genotoxicity of the fermentation products and whether these effects differ to those induced by addition of inulin. Although many proteins are largely digested before reaching the colon *in vivo*, the increase in colonic DNA damage in response to consumption of soy proteins suggests some may reach the colon and undergo fermentation, resulting in the production of genotoxic products. SCFA concentrations and bacterial numbers were also measured in the simulators during fermentations to determine whether they are associated with changes in genotoxicity.

## 2. Experimental Section

### 2.1. Faecal Inoculum

Stool from five healthy adult volunteers, who had not received antibiotics or experienced episodes of diarrhoea for 4 weeks prior to the study, were collected on two separate occasions and kept on ice for a maximum of 2 h at 5 °C in airtight plastic bags until use. The five samples were mixed together in an anaerobic chamber to eliminate inter-individual variation, and then homogenized and diluted in anaerobic PBS (0.1 M, pH 7.2) to produce a 20% (w v^−1^) faecal slurry. The slurry was prepared just prior to inoculation of the fermenter.

### 2.2. Fermentation Media

Fermentations were performed using a basal media prepared according to Bruck *et al.* [15] with minor changes: NaCl (0.05 g L^−1^, Sigma-Aldrich, Sydney, Australia), K_2_HPO4 (0.02 g L^−1^, BDH Laboratory Supplies, San Jose, CA, USA), KH_2_PO (0.02 g L^−1^, Ajax Finechem, Sydney, Australia), MgSO_4_·7H_2_O (0.005 g L^−1^, Sigma-Aldrich, Sydney, Australia), CaCl_2_·2H_2_O (0.0034 g L^−1^, BDH Laboratory Supplies, San Jose, CA, USA), NaHCO_3_ (1 g L^−1^, Sigma-Aldrich, Sydney, Australia), haemin (0.0025 g L^−1^, Sigma-Aldrich, Sydney, Australia), Cystein HCL (0.25 g L^−1^, Sigma-Aldrich, Sydney, Australia), bile salts (0.25 g L^−1^, Oxoid, Basingstoke, UK), Tween 80 (1 mL L^−1^, Sigma-Aldrich, Sydney, Australia) and vitamin K1 (5 µL L^−1^, Sigma-Aldrich, Sydney, Australia). The media was prepared in 10× stock solutions with pH adjusted to 7.0, sterilized by autoclaving at 121 °C for 20 min and kept at 5 °C. Media, ready for use in the fermenter, was prepared from stocks in a volume of 3 L just prior to connecting the media reservoir to the fermenter. The media was supplemented with 3% (w v^−1^) soy protein (Morlife Pty. Ltd., Labrador, Australia) previously shown to induce relatively high numbers of colonic DNA strand breaks when fed to rats at high levels [4]. Carbohydrate sources added to the fermenter, at 1% (w v^−1^), were a highly digestible low amylose cornstarch (3401C, The National Starch and Chemical Company, Sydney, Australia), xylo-oligosacharides (XOS) (DP 2-6; kindly provided by Danisco Health and Nutrition, Kantvik, Finland) or inulin (DP 2-60; Orafti ST-Gel, Beneo-Orafti, Tienen, Belgium).

### 2.3. Human Colonic Simulator

The fermenter was set up as described by Bruck *et al.* [15] with minor changes. The fermenter consisted of two glass vessels, a proximal vessel (PV) and a distal vessel (DV), with operating volumes of 220 mL and 320 mL, respectively. The vessels were connected by tubing and material from the PV supplied the DV. Vessel temperatures were kept at 37 °C and pH was automatically controlled with 0.1 M NaOH. The pH of the PV was kept at 5.5 and the DV at pH 6.8, both similar to their corresponding section of the human colon. Both vessels and the media reservoir were magnetically stirred and kept anaerobic by continuous gassing with sterile filtered oxygen-free nitrogen.

The fermenter was set up on the day prior to inoculation (Day 1). Basal media supplemented with 3% soy protein and 1% cornstarch was added to each vessel to allow anaerobic conditions to develop and the temperature to reach 37 °C. On the following day (Day 0) the faecal slurry was added to the vessels to a final concentration of 2% and the first sample (7 mL) was taken. On Day 1 the media reservoir and the pump were connected to the PV with a flow rate of 0.03 L h^−1^ (total transit time of 18 h). The PV subsequently supplied the DV. The media reservoir was changed every third day. Samples (7 mL) were taken daily in the morning from each vessel and kept at −80 °C until further analysis. The media contained low amylose cornstarch as the carbohydrate source for the first 10 days (days 1–10) followed by 10 days with XOS or inulin as carbohydrate source (days 11–20). The daily samples were used for SCFA measurements to assess the stability of the fermentation system which reached a steady state on day 6. Results reported are from days 8–10 (low amylose cornstarch) and days 18–20 (XOS or inulin) to ensure the fermentation is stabilised and the comparison between the low amylase starch and the prebiotic starch is within the same run.

### 2.4. Growth and Maintenance of HT29 Cells

Human HT29 colonocytes were used for assessment of fermenter genotoxicity. Cells were cultured in 75 cm^3^ tissue culture flasks (Greiner Bio-One, Frickenhausen, Germany) containing 20 mL of Dulbecco’s Modified Eagle’s Medium (DMEM) supplemented with 10% fetal bovine serum, 0.37% NaHCO_3_, 0.60% HEPES (Sigma-Aldrich, Sydney, Australia) and 1% antibiotic/antimycotic at 37 °C in an atmosphere of 5% CO_2_. All chemicals were from Invitrogen unless otherwise stated. pH of the media was adjusted to 7.3. Cells were passaged once weekly at ~90% confluence.

### 2.5. Faecal Water Genotoxicity Assay

The liquid phase in the PV and DV fermenter is referred to as faecal water. It was isolated from samples collected on Days 8, 9 and 10 (low amylose cornstarch) and Days 18, 19 and 20 (XOS or inulin) of fermentations in the PV and DV by centrifugation at 2000× g for 45 min at 4 °C and then stored at −80 °C. HT29 cells were treated with a homogeneous sample of faecal water from Days 8–10 combined or Days 18–20 combined. Media from a flask of ~90% confluent HT29 cells was carefully removed. Cells were washed twice in 10 mL pre-warmed (37 °C) PBS and incubated with 1 mL Trypsin-EDTA (Invitrogen, Carlsbad, NM, USA) for 5 min at 37 °C, 5% CO_2_. Cells were disaggregated in 10 mL pre-warmed (37 °C) supplemented DMEM, counted and diluted to a concentration of ~10^4^ cells mL^−1^. Cell suspensions (2 mL well^−1^) were added to 6-well tissue culture plates (BD Biosciences, San Jose, CA, USA) and incubated overnight at 37 °C, 5% CO_2_.

To test the effects of faecal water on the cultured cells, media was removed from cells growing overnight and cells were washed twice in 1 mL PBS. Faecal water was diluted to 20% with PBS and then 1 mL of the diluted faecal water was added to each well and cells were incubated for 30 min at 37 °C, 5% CO_2_. Previous validation of the model, testing 0.1% to 100% of faecal water, had shown 20% dilution of faecal water was an optimal concentration (data not shown). Control cells were incubated with 1 mL 50 μM H_2_O_2_ (positive control) (Sigma-Aldrich, Sydney, Australia) and with 1 mL PBS (negative control). After 30 min, solutions were removed and cells were washed twice in 1 mL PBS. Trypsin-EDTA (100 µL) was added to each well and plates were incubated for 5 min at 37 °C, 5% CO_2_. Pre-warmed DMEM supplemented with FBS (2 mL) was added to each well and cells were carefully disaggregated. From each well 500 µL aliquots were transferred to microcentrifuge tubes, and centrifuged at 4000 rpm for 5 min. Media was discarded and cells were then analysed by the comet assay. Trypan blue was added to 20 µL cell suspensions from each well to determine the viability of cells (~95% viability was achieved).

### 2.6. Comet Assay

DNA single-strand breaks (SSB) induced by the faecal water treatments were investigated using the single-cell gel electrophoresis assay (comet assay) [16,17]. Cells isolated from the faecal water assay were resuspended in 200 µL pre-warmed low melting-point agarose (LMA) (Trevigen, Gaithersburg, MD, USA) and 45 µL was pipetted onto the 1st well of two comet assay glass slides (Trevigen, Gaithersburg, MD, USA). Another 45 µL LMA was added to the remaining cells, mixed briefly and 45 µL was pipetted onto the 2nd well of each slide. The cell suspension was spread evenly across the surface of the glass slide, covered with a cover slip and kept on ice for 30 min to allow the agarose to solidify. The cover slips were removed and the slides were immersed in a cold lysis buffer (Trevigen, Gaithersburg, MD, USA) at 4 °C for 1 h. Slides were placed in an electrophoresis tank containing alkaline electrophoresis buffer (300 mM NaOH, 1 mM Na_2_EDTA, pH > 13) kept at 4 °C. Slides were submerged in the buffer for 20 min before electrophoresis were conducted at 25 V, 300 mM for 20 min. Slides were removed from the alkaline buffer and placed in pH neutralizing buffer (400 mM Tris-HCl, pH 7.5) three times (5 min each time), fixed in 96% ethanol for 5 min and left to dry at 37 °C for 5 min. Slides were stained with propidium iodide (Sigma-Aldrich, Sydney, Australia) and images were captured using an Olympus BX-41 fluorescence microscope and the software Image Pro Plus (Media Cybernetics Inc., Rockville, MD, USA). Tail length and comet tail moment (the product of tail length and the fraction of DNA in the tail) were calculated for ~50 cells per slide using CometScore™ v1.5 (TriTek Corp., Sumerduck, VA, USA). Apoptotic cells were excluded from the analysis based on their morphology.

### 2.7. SCFA Analysis

Fermentation samples (1 mL) from days 1–20 were diluted 1:3 with a solution of 1.68 mM Heptanoic acid (internal standard), pH 7, and left for sedimentation of particulate material. Supernatants (150 µL) were distilled by vacuum distillation as described by McOrist, *et al*. [18]. Distillates (60 µL) were analysed for SCFA (acetate, butyrate and propionate) in duplicate using an Agilent Technologies 6890N Network Gas Chromatograph System fitted with a Zebron ZB-FFAP capillary GC column (Dimension: 30 m × 0.53 mm I.D.) (Phenomenex, Torrance, CA, USA) as previously described by McOrist *et al*. [18]. SCFA results from days 8–10 (low amylose cornstarch) and days 18–20 (inulin or XOS) were averaged.

### 2.8. DNA Extraction and Quantitative PCR (QPCR)

DNA was extracted from 0.5 mL of the fermentation samples collected on Days 8–10 (low amylose cornstarch) and Days 18–20 (XOS and inulin). Samples were centrifuged at 13,000× g for 5 min and pellets were resuspended in 1.2 mL TE Buffer (10 mM Tris-HCl, 1 mM EDTA, pH 8) and transferred to 2 mL microcentrifuge tubes containing 0.5 mL zirconia-silica beads (0.1 mm, Biospec Products) and 30 µL 10% SDS. Bacteria cells were lysed by shaking for 5 min on a minibead beater on high speed and centrifuged at 4500 g for 1 min. DNA was extracted from the supernatant using the QIAamp DNA Stool Mini Kit (Qiagen, Doncaster, Victoria, Australia) according to the manufacturer’s instructions and stored at −20 °C until use. DNA concentrations were quantified using Quanti-iT Pico Green (Invitrogen, Carlsbad, NM, USA) with fluorescence measured using a PTC-200 Peltier Thermal Cycler (MJ Research Inc., Waltham, MA, USA).

Quantitative PCR was performed on DNA extracted from fermentation samples collected on Days 8–10 (low amylose cornstarch) and Days 18–20 (XOS and inulin). Primers and amplification conditions for quantification of specific bacterial groups and species are listed in Table 1. All reactions were performed in 10 µL reactions with 1 µL template DNA (10 ng µL^−1^), except *Akkermansia municiphila* and sulphate-reducing bacteria (SRB). For these assays 3 μL template DNA (10 ng μL^−1^) was used in a 20 μL reaction. Each reaction contained template DNA, Ssofast Evagreen Supermix (2×) (Bio-Rad), primers (Table 1), 0.4 μL BSA (Promega, Sydney, Australia) or 1 μL Dimethyl Sulfoxide (SRB only) (Sigma-Aldrich, Sydney, Australia) and Milli-Q to a final volume of 10 or 20 μL. Each sample was analysed in triplicates per PCR assay. Amplifications were performed with an initial denaturation at 98 °C for 3 min. followed by 35–40 cycles of 98 °C for 15 s, 58–65 °C for 15–60 s and 72 °C for 30–60 s (Table 1). A final melting-curve analysis was performed after completion of all cycles with fluorescence collected at 0.5 °C intervals between 55 and 95 °C. An 8-series of 10-fold dilutions of a sample derived plasmid construct (Topo chemical competent cells, Invitrogen, Carlsbad, NM, USA) containing the target amplicon were analysed in parallel with DNA samples for estimation of PCR efficiency for all assays. All reactions were run on a PTC-200 Peltier Thermal Cycler (MJ Research Inc., Waltham, MA, USA) and analysed using MJ Opticon Monitor Analysis Software Version 3.1 (Bio-Rad Laboratories, Hercules, CA, USA). For estimation of relative fold change of bacterial groups results were analysed with qBase^+^ (Biogazelle, Gent, Belgium) [19,20]. The data was normalised to the total number of bacteria for each sample and expressed as fold change compared to the low amylose cornstarch treatment in each vessel for each substrate. All calculations were done using an assay specific PCR efficiency.

**Table 1:** Primers and amplification conditions for real-time PCR assays.

Target	Primer	Sequence (5′–3′)	nM	Reference	Annealing		Elongation	
					°C	Time (s)	°C	Time (s)
Total bacteria	1114F 1275R	CGGCAACGAGCGCAACCC CCATTGTAGCACGTGTGTAGCC	150	[21]	60	20	72	45
*Bacteroides fragilis* group	Bfr-F Bfr-R	CTGAACCAGCCAAGTAGCG CCGCAAACTTTCACAACTGACTTA	500	[22]	58	60	72	30
*Bifidobacterium* spp.	Bif-F Bif-R	TCGCGTC(C/T)GGTGTGAAAG CCACATCCAGC(A/G)TCCAC	600	[23]	58	20	72	30
*Clostridium* *coccoides* group	g-Ccoc-F g-Ccoc-R	AAATGACGGTACCTGACTAA CTTTGAGTTTCATTCTTGCGA A	250	[24]	58	20	72	45
*Clostridium* *leptum* group	sg-Clept-F sg-Clept-R	CTTTGAGTTTCATTCTTGCGAA GCACAAGCAGTGGAGT	250	[24]	58	20	72	45
*Escherichia* *coli*	E.coli F E.coli R	CATGCCGCGTGTATGAAGAA CGGGTAACGTCAATGAGCAAA	375	[25]	60	20	72	45
*Faecalibacterium praunitzii*	FPR-1F FPR-2R	AGATGGCCTCGCGTCCGA CCGAAGACCTTCTTCCTCC	500	[26]	62	20	72	40
*Lactobacillus* group	Lacto-F Lacto-R	AGCAGTAGGGAATCTTCCA CACCGCTACACATGGAG	500	[27] [28]	58	30	72	30
SRB ^1^_*aps* ^2^	APS3F APS2R	TGGCAGATCATGWTYAAYGG GGGCCGTAACCRTCYTTRAA	400	[29]	58	30	72	60
SRB_*dsr* ^3^	DSR1F+ DSR-R	ACSCACTGGAAGCACGGCGG GTGGMRCCGTGCAKRTTGG	400	[30]	65	15	72	30

^1^ Sulfate-reducing bacteria; ^2^ Adenosine-5-phosphosulfate reductase gene; ^3^ Dissimilatory sulfite reductase gene.

### 2.9. Statistics

SCFA data and DNA damage data was tested for normal distribution using the Shapiro-Wilk test. Non-normal distributed data was transformed, propionate concentrations were transformed using natural logarithm, whereas tail length was transformed using reflect and square root. Normal and transformed data were analysed using a one-way analysis of variance (ANOVA) to assess significant difference between groups within fermenter and treatment. PASW version 18 [31] computer software was used for these statistical analyses. The microbial data was normalised to the total number of bacteria in the sample and presented as fold change relative to the low amylose cornstarch treatment for the appropriate vessel and substrate. Microbial fold changes were analysed for changes within vessels between inulin and XOS, and between vessels within substrate using the multivariate statistical package Primer 6 with Permanova^+^ (PRIMER-E Ltd., Plymouth, UK) using one- and two-factorial analysis based on a Euclidean distance matrix. Distance-based linear models (DISTLM) and distance based redundancy analysis (dbRDA) [32,33] was used to investigate the relationship between microbial data, DNA damage and biochemical measures To identify the key biochemical measures best explaining the relationship between the different data *Best* was used as selection procedure and adjusted *R*^2^ as the selection criteria. A *P*-value of less than 0.05 was considered statistical significant.

## 3. Results

### 3.1. Faecal Water Genotoxicity

There were no differences in the effect of inulin fermentation on the genotoxicity of faecal water samples from the two vessels when compared to fermentation with cornstarch (Table 2). The fermentation of XOS resulted in a significant decrease in faecal water genotoxicity in the PV compared to the effect of cornstarch fermentation (Table 2). In contrast, a significant increase in faecal water genotoxicity was observed in the DV (Table 2).

### 3.2. Short Chain Fatty Acids

Inulin and XOS significantly increased the concentration of butyrate, but no other major SCFA, in the DV relative to the concentrations resulting from fermentation of cornstarch (Table 3). No significant effects on SCFA were detected in the PV.

**Table 2:** Faecal water genotoxicity of low amylose cornstarch, inulin and XOS in a human colonic simulator. Genotoxicity (mean ± SEM) was measured as numbers of DNA single strand breaks in cultured colonic cells using the comet assay following incubation of the cells with faecal water. Data are presented as tail moment and tail length. PV: proximal vessel; DV: distal vessel.

	PV (pH 5.5)				DV (pH 6.8)				PBS (control)
	Pre-inulin	Inulin	Pre-XOS	XOS	Pre-inulin	Inulin	Pre-XOS	XOS	
Tail Moment	18.6 ± 1.1	16.0 ± 0.7	**7.6 ± 0.4** ^a^	**5.4 ± 0.3** ^b^	11.3 ± 0.5	12.1 ± 0.6	**6.7 ± 0.3** ^b^	**12.8 ± 0.6** ^a^	5.8 ± 0.7
Tail Length	78.9 ± 3.7	76.7 ± 2.8	**44.1 ± 1.7** ^a^	**33.3 ± 1.4** ^b^	54.9 ± 1.9	61.2 ± 2.5	**34.5 ± 1.2** ^b^	**62.0 ± 2.2** ^a^	34.3 ± 3.1

Means of treatment pairs with unlike superscript letters are significantly different (ANOVA; *p* < 0.05).

**Table 3:** Short chain fatty acid (SCFA) concentrations. SCFA (µmoL/g) are presented as the mean ± SEM and measured in vessels after fermentation of low amylose cornstarch (pre-inulin and pre-XOS), inulin and XOS in a human colonic simulator. PV: proximal vessel; DV: distal vessel.

SCFA	PV (pH 5.5)				DV (pH 6.8)			
	Pre-inulin	Inulin	Pre-XOS	XOS	Pre-inulin	Inulin	Pre-XOS	XOS
Acetate	43 ± 7	47 ± 6	84 ± 4	86 ± 7	95 ± 12	97 ± 9	110 ± 8	104 ± 3
Propionate	1 ± 0	1 ± 0	3 ± 1	3 ± 1	19 ± 1	22 ± 4	21 ± 2	22 ± 4
Butyrate	28 ± 4	37 ± 4	15 ± 2	24 ± 6	**39 ± 5 ^a^**	**70 ± 6 ^b^**	**35 ± 2 ^a^**	**46 ± 6 ^b^**
Total	72 ± 11	85 ± 6	102 ± 6	113 ± 13	153 ± 18	189 ± 14	178 ± 12	181 ± 14

Means of treatment pairs with unlike superscript letters are significantly different (ANOVA; *P* < 0.05).

### 3.3. Microbial Abundances

**Figure 1:**
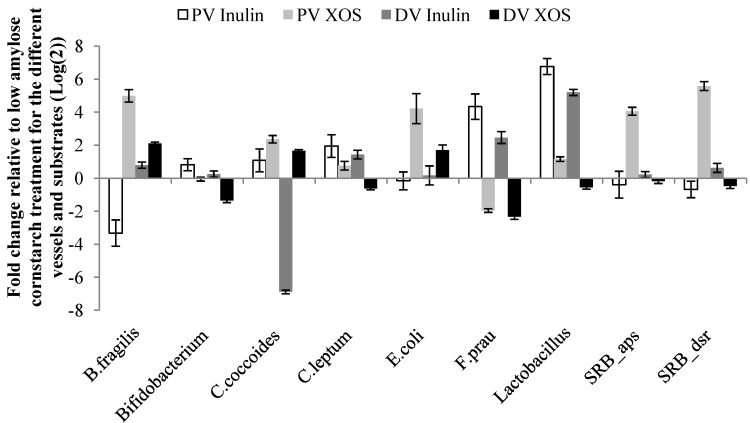
Microbial response to changes in carbohydrate source in a human colonic simulator. Changes are presented as fold change (mean ± SEM) relative to low amylose cornstarch treatment for the two vessels and inulin or XOS, respectively. PV: proximal vessel; DV: distal vessel. SRB: Sulfate-reducing bacteria, *aps*: Adenosine-5-phosphosulfate reductase gene, *dsr*: Dissimilatory sulfite reductase gene.

Bacterial populations were analysed for differences between substrates within each vessel and between vessels within substrate. In the PV, the *Bacteroides fragilis* group responded significantly differently to XOS and inulin as it increased in response to XOS but decreased in numbers when inulin was fermented (Figure 1 and Table 4). Although, inulin stimulated growth in the DV the relative abundance of the *B. fragilis* group was significant higher with XOS (Figure 1 and Table 4). Populations of the *B. fragilis* group also differed significantly between the two vessels for both XOS and inulin. *Bifidobacterium* spp. were increased in both the PV and DV after inulin fermentation and decreased in the DV after XOS fermentation (Figure 1 and Table 4). This resulted in a significant higher abundance of *Bifidobacterium* spp. with inulin in both PV and DV, when substrates were compared within vessels. In the DV, the relative abundance of the *Clostridium coccoides* group was decreased following inulin fermentation leading to a significant difference between inulin and XOS. Both substrates also significantly decreased the relative abundance of the *C. coccoides* group from PV to DV (Figure 1 and Table 4). The *Clostridium leptum* group was significantly reduced in the DV after XOS fermentation this lead to a significant difference between substrates. XOS also resulted in a significant difference in these bacteria between the PV and DV (Figure 1 and Table 4). The relative fold change of *Escherichia coli* spp. was significantly higher for XOS than inulin in the PV, this was also the case in the DV. Therefore, the relative fold change of *E. coli* spp. significantly decreased between PV and DV during XOS fermentation but not with inulin fermentation. In the PV and DV, *Faecalibacterium prausnitzii* was significantly stimulated by inulin but inhibited by XOS (Figure 1 and Table 4), but the increase was greater in the PV compared to the DV for inulin which meant a significant decrease in *F. prausnitzii* between the PV and DV for inulin. *Lactobacillus* spp. was greatly stimulated by inulin and only limited by XOS in the PV resulting in a significant difference between the two carbohydrates. This significance was carried over into the DV, however, *Lactobacillus* spp. were reduced by XOS. The relative fold change of *Lactobacillus* spp. was also significantly different between vessels for both substrates. SRB measured using the adenosine-5-phosphosulfate reductase gene (*aps*) and the dissimilatory sulfite reductase gene (*dsr*) both showed a significant reduction in SRB relative fold change with inulin and a significant increase with XOS in the PV. In the DV, only *dsr* showed a significant difference between substrates. Both assays also showed a significant difference between PV and DV for XOS (Figure 1 and Table 4).

### 3.4. Relationships between Microbial Abundance, DNA Damage and SCFA

**Figure 2:**
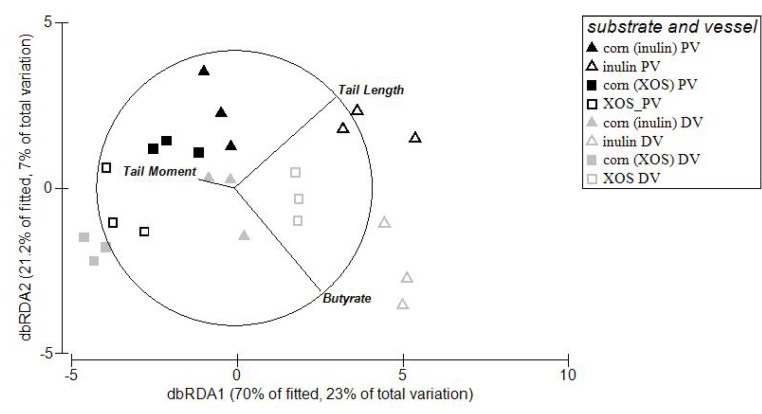
Distribution of the variation in the relative microbial abundance displayed based on a distance based redundancy analysis (dbRDA) with vectors indicating the weight and direction of the best predictor variables from the biochemical results.PV: proximal vessel; DV: distal vessel.

The relationships between the abundance of microbes and levels of DNA damage and SCFA were investigated. Three variables had a significant relationship with the microbial data (Tail moment: *p* = 0.027; Tail length: *p* = 0.001; Butyrate: *p* = 0.011) (Figure 2). Correlation analyses showed that acetate was negatively correlated (Pearson’s correlation coefficient (*R*)) with both tail length (*R*: −0.66; *p* < 0.001) and tail moment (*R*: −0.67; *p* < 0.001). DNA damage was also negatively correlated with the relative abundance of *B. fragilis* (*R*: −0.49; *p* = 0.014), SRB_*aps* (*R*: −0.41; *p* = 0.044) and SRB_*dsr* (*R*: −0.41; *p* = 0.048).

**Table 4:** Statistical differences (*p* < 0.05) between substrates within individual vessels and for substrates between vessels for each bacterial group/species. PV: proximal vessel; DV: distal vessel. SRB: Sulfate-reducing bacteria, *aps*: Adenosine-5-phosphosulfate reductase gene, *dsr*: Dissimilatory sulfite reductase gene.

Comparisons	*B.* *fragilis*	*Bifidobacterium*	*C.* *coccoides*	*C.* *leptum*	*E.* *coli*	*F.* *prau*	*Lactobacillus*	SRB_*aps*	SRB_*dsr*
PV inulin *vs.* PV XOS	˃0.0001	0.02	*ns*	*ns*	0.0007	˃0.0001	˃0.0001	0.0002	˃0.0001
PV inulin *vs.* DV inulin	0.003	*ns*	˃0.0001	*ns*	*ns*	0.038	0.007	*ns*	0.046
PV XOS *vs.* DV XOS	˃0.0001	˃0.0001	0.008	0.0002	0.012	*ns*	˃0.0001	˃0.0001	˃0.0001
DV XOS *vs.* DV inulin	˃0.0001	˃0.0001	˃0.0001	0.0001	0.030	˃0.0001	˃0.0001	*ns*	0.002

*ns*: no significance.

## 4. Discussion

The present study investigated the effects of adding XOS or inulin to the fermentation of soy protein by a mixed faecal microbiota in vessels of a human colonic simulator that approximate the pH of the proximal and distal colon. We have demonstrated that XOS, but not inulin, can significantly influence the genotoxicity of the fermentation products using this *in vitro* system and those differences may be related to differences in the microbiota. The lowering of the genotoxicity of PV contents by XOS fermentation suggests that addition of this oligosaccharide to the diet in humans could protect against high dietary protein-induced damage to tissues of the proximal colon. However, in contrast to the XOS-induced reduction in genotoxicity in the PV, XOS increased genotoxicity in the DV. In humans, tumors occur more frequently in the distal region of the colon than the proximal region and it is possible that the pH of the region is an important determinant of the genotoxicity of bacterial fermentation products.

Differences in the genotoxicity between vessels and carbohydrate treatments could be explained by changes in the microbiota. The *B. fragilis* group, *E. coli* and SRB were stimulated by XOS fermentation in the PV whereas for inulin these bacteria were reduced or remained at similar levels. Correlation between variables found a significant inverse relationship between the abundances of *B. fragilis*, SRB and the DNA SSB that resulted from incubation of the colonic cells with fecal water, suggesting a protective role of these bacteria in this study. Bacteria belonging to the *B. fragilis* group possess the ability to utilise polysaccharides, and a recent study by Petersen *et al.* [34] demonstrated increased levels of the *B. fragilis* group in the faecal microbiota of mice fed XOS. Our data showing an increase in numbers of the *B. fragilis* group in the PV in response to XOS supports this *in vivo* data.

The role of SRB in gastrointestinal health is poorly understood and remains unclear [35] but H_2_S produced by these bacteria can be toxic to the gut epithelium [36]. SRB could contribute to the pathogenesis of colorectal diseases, such as inflammatory bowel disease [37], and evidence to date indicates that the role of faecal H_2_S concentrations and colonic *Desulfovibrio* spp. also remains unclear in ulcerative colitis patients [38,39,40,41]. However, the abundance of *Desulfovibrio* spp. reported in some of these studies has to be interpreted with caution, as the PCR primers used overestimate SRB abundance [29]. In contrast, gene-specific primers targeting the Adenosine-5-phosphosulfate reductase gene and the dissimilatory sulfite reductase gene, and not 16S rRNA primers, were used in this study. Our results do not rule out a positive role for SRB in the gut. Other studies support a beneficial role for H_2_S. Wallace *et al.* [42] have shown that endogenous H_2_S prevents experimentally-induced colitis in rats, suggesting an anti-inflammatory effect.

The present study did not demonstrate a bifidogenic effect of XOS or inulin as seen in previous studies [12,34,43,44]. However, changes in *Lactobacillus* spp. occurred as expected, with inulin increasing the *Lactobacillus* spp. and XOS not having a real effect [34,45,46].

There is a growing body of evidence that the SCFA, particularly butyrate, are key mediators of the bowel health benefits of dietary fibre [3,47,48,49], although products of bacterial fermentation other than SCFA could also contribute to the health of the bowel. In the present study, a significant elevation in the concentration of butyrate, but no other SCFA, was seen in the DV for both XOS and inulin when compared with low amylose cornstarch but this did not translate to a lower genotoxicity of the content of these vessels. This suggested another factor was the primary contributor to differences in genotoxicity. Further analysis of the relationships between variables showed there was a strong inverse correlation between acetate and the faecal water genotoxicity. A similar observation has been made previously by Toden *et al*. [3] in a study examining the effects of RS on colonic DNA damage in rats. Acetate has also been shown to help protect against enteropathogenic infections [14] and a similar protection of the HT-29 cells may occur in our faecal water genotoxicity assay. As acetate is the most abundant of the SCFA produced in the gut its actions are often thought to be related to its capacity to lower pH, but that cannot be the case in this study as pH was kept constant in the two vessels. This and other studies [3,14] indicate that acetate may have a direct effect on protection against DNA damage, whereas butyrate has a secondary effect through its ability to repair DNA damage. However, the relationship between acetate and DNA damage needs further investigation.

The increase in butyrate concentrations in response to XOS and inulin would be expected to be related to changes in bacterial populations and/or their metabolic activity, including increased numbers of butyrate-producers. In humans, some of the most numerically abundant butyrate-producers are *Roseburia* spp. and *Eubacterium rectale*, both members of the *C. coccoides* group, and *F. prausnitzii*, which belongs to the *C. leptum* group [49]. The current study indicates that the increases in DV butyrate levels for inulin and XOS were likely to come from different groups of bacteria. Inulin increased bacteria of the *C. leptum* group and *F. prausnitzii*, as seen in the study of Ramirez-Farias *et al*. [12], but decreased the *C. coccoides* group in the DV. This was the opposite with XOS fermentation which increased numbers of the *C. coccoides* group and lowered numbers of *F. prausnitzii* and the *C. leptum* group. This difference could be due to differences in length and structure of the two carbohydrates.

## 5. Conclusions

The addition of XOS to a simulator of human colonic fermentation has demonstrated that it can alter the genotoxicity of fermented material in the presence of soy protein. The decrease in genotoxicity within the PV suggests dietary XOS could potentially lower protein-induced proximal colon genotoxicity and tissue damage in humans. The effects of XOS but not inulin on genotoxicity may be related to observed differences in effects of these substrates on the microbiota. Different populations of bacteria seem to be responsible for increased production of butyrate by inulin and XOS. This study also indicates a role for acetate in relation to protection against DNA damage, but that relationship requires further investigation.
